# The Protective Role of 1,8-Dihydroxynaphthalene–Melanin on Conidia of the Opportunistic Human Pathogen Aspergillus fumigatus Revisited: No Role in Protection against Hydrogen Peroxide and Superoxides

**DOI:** 10.1128/msphere.00874-21

**Published:** 2022-01-05

**Authors:** E. M. Keizer, I. D. Valdes, B. L. McCann, E. M. Bignell, H. A. B. Wösten, H. de Cock

**Affiliations:** a Microbiology, Department of Biology, Utrecht University, Utrecht, the Netherlands; b Institute of Biomembranes, Utrecht University, Utrecht, the Netherlands; c MRC Centre for Medical Mycology, University of Exeter, Exerter, United Kingdom; University of Georgia

**Keywords:** *Aspergillus fumigatus*, DHN-melanin, catalase, conidia, hydrogen peroxide, reactive oxygen species

## Abstract

Previously, 1,8-dihydroxynaphthalene (DHN)-melanin was described to protect Aspergillus fumigatus against hydrogen peroxide (H_2_O_2_), thereby protecting this opportunistic human pathogen from reactive oxygen species generated by the immune system. This was based on the finding that the ATCC 46645 mutant with mutations in the *pksP* gene of the DHN-melanin synthesis pathway showed increased sensitivity to reactive oxygen species compared to the wild type. Here, it is shown that deletion of the *pksP* gene in A. fumigatus strain CEA10 did not affect sensitivity for H_2_O_2_ and superoxide in a plate stress assay. In addition, direct exposure of the dormant white conidia of the *pksP* deletion strains to H_2_O_2_ did not result in increased sensitivity. Moreover, complementation of the ATCC 46645 *pksP* mutant strain with the wild-type *pksP* gene did result in pigmented conidia but did not rescue the H_2_O_2_-sensitive phenotype observed in the plate stress assay. Genome sequencing of the ATCC 46645 *pksP* mutant strain and its complemented strain revealed a mutation in the *cat1* gene, likely due to the UV mutagenesis procedure used previously, which could explain the increased sensitivity toward H_2_O_2_. In summary, DHN-melanin is not involved in protection against H_2_O_2_ or superoxide and, thus, has no role in survival of conidia when attacked by these reactive oxygen species.

**IMPORTANCE** Opportunistic pathogens like Aspergillus fumigatus have strategies to protect themselves against reactive oxygen species like hydrogen peroxides and superoxides that are produced by immune cells. DHN-melanin is the green pigment on conidia of Aspergillus fumigatus and more than 2 decades ago was reported to protect conidia against hydrogen peroxide. Here, we correct this misinterpretation by showing that DHN-melanin actually is not involved in protection of conidia against hydrogen peroxide. We show that UV mutagenesis that was previously used to select a *pksP* mutant generated many more genome-wide mutations. We discovered that a mutation in the mycelial catalase gene *cat1* could explain the observed phenotype of increased hydrogen peroxide sensitivity. Our work shows that UV mutagenesis is not the preferred methodology to be used for generating mutants. It requires genome sequencing with single-nucleotide polymorphism analysis as well as additional validations to discard unwanted and confirm correct phenotypes.

## INTRODUCTION

Aspergillus fumigatus is a saprotrophic fungus that feeds on organic material of either dead or living organisms (1). Asexual reproduction generates massive amounts of conidia that are dispersed into the air. Humans inhale several hundred A. fumigatus conidia daily (2), and due to their small size (<5 μm) (3), inhaled conidia can reach the deeper parts of the respiratory tract (4). Here, they can cause noninvasive or invasive infections in especially immunocompromised patients (5).

Reactive oxygen species (ROS) are oxygen-derived molecules that include radicals like superoxide (O_2_^−^), hydrogen peroxide (H_2_O_2_), and hydroxyl radicals (^•^OH) but also nonradicals like hydrochlorous acid and ozone (6). These molecules play an important role in immune defense, and genetic defects affecting ROS production in individuals compromises health (7). ROS damage many cellular components, including DNA and proteins, and drive lipid peroxidation (8). Phagocyte-derived ROS are produced by the NADPH-oxidase complexes that are part of the NOX family (6). These complexes are also expressed in type II epithelial and ciliated lung cells (9). A NOX-mediated ROS release, referred to as an oxidative burst, along with reactive nitrogen species enables microbial killing (10). The importance of the oxidative burst is shown in patients with the chronic granulomatous disease (CGD), which is caused by various defects in components of the NOX2 enzyme complex. CGD patients are characterized by recurrent fungal and bacterial infections (11). CGD mouse models also show high susceptibility to A. fumigatus infections (12). Besides defense against microbial pathogens, ROS also serve as messengers for the innate and adaptive immune response (13). Activation of TLR1, TLR2, and TLR4 requires ROS for optimal activity in macrophages (14). The activation of the adaptive immune response by B and T cells is dependent on the production of ROS (15, 16).

The green pigment 1,8-dihydroxynaphthalene (DHN)-melanin was reported to protect A. fumigatus conidia against peroxide (17). However, deletion of *abr2*, involved in early steps in the DHN-melanin pathway, did not alter peroxide sensitivity compared to the wild-type (WT) strain. This indicates that intermediates of DHN-melanin are sufficient to protect the conidia against peroxide (18). l-DOPA melanin was shown to protect against hydroxyl radicals in the human-pathogenic dimorphic fungus Cryptococcus neoformans (19). Aspergillus nidulans also produces l-DOPA melanin (20). In this fungus, deletion of the polyketide synthase gene *wA* stops the production of l-DOPA melanin and leads to increased sensitivity to H_2_O_2_ (21).

As with melanin, fungi have different types of enzymes that protect against ROS. Superoxides are converted to H_2_O_2_ by superoxide dismutases (SODs). The *sod1*-s*od4* genes of A. fumigatus encode such enzymes. Deletion of s*od1* and *sod2* (in a CEA10 genetic background) leads to increased sensitivity to superoxides. Deletion of *sod3* (in a CEA10 genetic background) does not alter the sensitivity to superoxides, while the role of *sod4* needs to be determined, as it is an essential gene and therefore could not be deleted (22). H_2_O_2_ is converted to water and oxygen by enzymes such as catalases, peroxidases, peroxiredoxins, and the thioredoxin system (23). There are three different catalase genes known in A. fumigatus, the conidial catalase *catA* and the two mycelial catalases *cat1* and *cat2*. Deletion of *catA* (in a CEA10 genetic background) increases the sensitivity of the conidia to H_2_O_2_ but does not alter killing by murine alveolar macrophages (24). Deletion of *cat1* (in a CEA10 genetic background) does not alter the sensitivity of germinated conidia of the fungus to H_2_O_2_, as determined in a metabolic assay (25), and does not affect survival of conidia in a direct H_2_O_2_ exposure assay or during exposure to polymorphonuclear (PMN) cells (24). Similarly, mycelia of a *cat2* deletion strain (in a CEA10 genetic background) do not show increased sensitivity to direct exposure of H_2_O_2_. However, mycelia of a *cat1-cat2* (in a CEA10 genetic background) double deletion strain are slightly more sensitive for H_2_O_2_, although no difference in killing by PMN cells is observed relative to the wild-type progenitor. Nonetheless, the double deletion mutant develops slower in lungs of infected rats (24).

The *prx1*, *prxB*, and *prxC* genes of A. fumigatus encode peroxiredoxins present in the cytosol (Prx1) or the mitochondria (PrxB and PrxC). Purified Prx1 and PrxC can decompose H_2_O_2_ (26). Deletion of each of these three genes in A. fumigatus (in a CEA10 genetic background) increases sensitivity to paraquat and menadione that generate H_2_O_2_ and superoxides via redox cycling, with the Δ*prx1* strain being most sensitive. Remarkably, the three single-deletion strains do not show increased sensitivity to H_2_O_2_ in a plate stress assay (26). In contrast, deletion of the fungal allergen peroxiredoxin Asp f3 (in a D141 genetic background) does result in increased H_2_O_2_ sensitivity, as determined in a plate stress assay (27). Absence of Asp f3 also results in an increased sensitivity for superoxides and inhibited hyphal growth. Furthermore, Asp f3 is essential for virulence in an immunocompromised mouse model (27).

As the role of melanin in the protection against H_2_O_2_ in A. fumigatus ATCC 46645 is well documented in previous research, we realized that this work was based on a *pksP* mutant generated by UV mutagenesis that can introduce additional mutations that might affect peroxide sensitivity. Furthermore, no complementation of this *pksP* mutant strain was performed. In this study, we wanted to confirm the role of DHN-melanin in protection against H_2_O_2_ in A. fumigatus. Next to the role of DHN-melanin in protection against H_2_O_2_, superoxides generated via menadione also were included to see the protective role of DHN-melanin against this other type of ROS. We now show that deletion of the *pksP* gene in A. fumigatus strain CEA10 did not affect sensitivity for H_2_O_2_ and superoxide in a plate stress assay, in contrast to the ATCC 46645 *pksP* mutant strain that was generated by UV mutagenesis (17, 18). Complementation of the latter strain with the intact *pksP* gene did not rescue the phenotype. Whole-genome sequencing and single-nucleotide polymorphism (SNP) analysis showed that the increase in H_2_O_2_ sensitivity of the ATCC 46645 *pksP* mutant might be due to a mutation in the *cat1* gene. Together, here we show for the first time that DHN-melanin is not involved in protecting conidia against hydrogen peroxide and superoxide.

## RESULTS

### Sensitivity of DHN-melanin mutants to oxidative stress.

**FIG 1:**
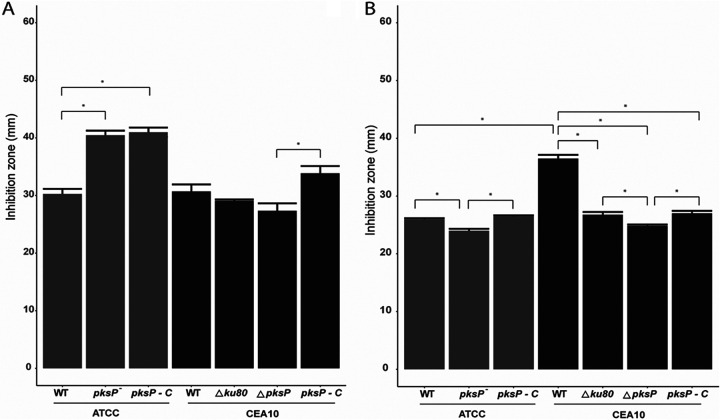
Peroxide (A) and menadione (B) sensitivity of A. fumigatus strains in the ATCC 46645 background with the wild-type, *pksP^−^* mutant, and complementation strains (gray) and the CEA10 background with the Δ*ku80*, Δ*pksp*, and complementation strains (black). Bars represent the average inhibition zone based on biological and technical triplicates with standard errors. Representative images of the plates can be found in Fig. S4.

Conidia of the ATCC 46645 UV mutant strain that contains mutations in the *pksP* gene are white and thereby do not produce melanin. These conidia were more sensitive to H_2_O_2_ than its parental WT strain in the H_2_O_2_ plate stress assay (Fig. 1A, gray bars), which is in accordance with reference 18. Deletion of the *pksP* gene by replacing the gene with the hygromycin resistance cassette in the CEA10Δ*ku80* background also resulted in a white phenotype but did not lead to an increased sensitivity toward H_2_O_2_ (Fig. 1A, black bars). Notably, complementation of the mutated *pksP* gene in the ATCC 46645 UV mutant resulted in green conidia but did not rescue the H_2_O_2_ sensitivity to wild-type levels. The complemented ATCC 46645 strain was as sensitive as the *pksP* mutant strain. In contrast, complementation of the CEA10Δ*ku80 pksP* deletion strain with a WT *pksP* gene resulted in green conidia, and the strain was still as resistant to H_2_O_2_ as the WT strain. These results show that DHN-melanin is not involved in protection against H_2_O_2_, as was previously concluded (17). Indeed, deletion of *pksA* resulting in a DHN-melanin-deficient strain in *P. roqueforti* resulted in white conidia (28) but again not in altered sensitivity to H_2_O_2_ in the plate assay (Fig. S5).

10.1128/msphere.00874-21.7 FIG S5 Peroxide sensitivity of *P. roqueforti* with or without (Δ*pks*) DHN-melanin based on inhibition zones. Bars represent the average inhibition zone based on biological and technical triplicates, with standard errors. Download FIG S5, TIF file, 0.1 MB. Copyright © 2022 Keizer et al. 2022 Keizer et al. https://creativecommons.org/licenses/by/4.0/ This content is distributed under the terms of the Creative Commons Attribution 4.0 International license.

**FIG 2:**
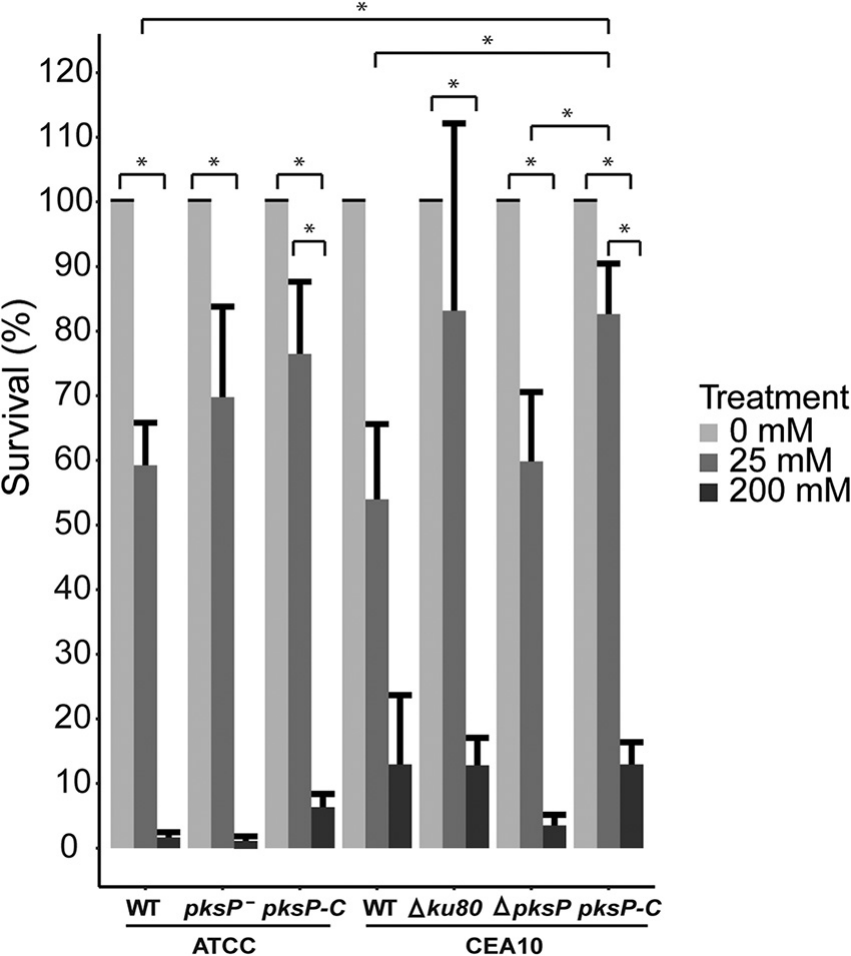
Sensitivity of dormant conidia to 0 mM, 25 mM, or 200 mM H_2_O_2_. Bars represent the average survival based on biological triplicates with the standard errors.

Dormant conidia were exposed for 30 min to 0, 25, and 200 mM H_2_O_2_. A decrease in survival of conidia was observed for all strains when the concentration of H_2_O_2_ was increased from 25 mM to 200 mM (Fig. 2). Deletion or complementation of the *pksP* gene in the CEA10 background, mutations in the *pksP* gene, and complementation in the ATCC 46645 background did not significantly alter the sensitivity of dormant conidia to the different concentrations of H_2_O_2_ (Fig. 2). Together, the removal or complementation of DHN-melanin in an ATCC or CEA10 background did not alter the sensitivity of dormant conidia to direct H_2_O_2_ exposure.

We next investigated sensitivity of DHN-melanin mutants to menadione, which induces production of superoxides (29). The ATCC 46645 *pksP^−^* mutant and its complemented strain did not show altered resistance to menadione compared to the WT strain (Fig. 1B, gray bars). The CEA10 wild-type strain appeared more sensitive to menadione than the ATCC 46645 wild-type strain. Interestingly, deletion of the *ku80* gene in the CEA10 WT strain resulted in an increased resistance to menadione. Deletion of the *pksP* gene also increased the resistance to menadione in the CEA10 background. On the other hand, complementation of the *pksP* gene decreased resistance to the same level as the CEA10Δ*ku80* strain (Fig. 1B, black bars). Together, these results show that DHN melanin is not involved in resistance to superoxides. In fact, it slightly increases sensitivity.

### SNPs underlying melanin deficiency and H_2_O_2_ sensitivity.

**FIG 3:**
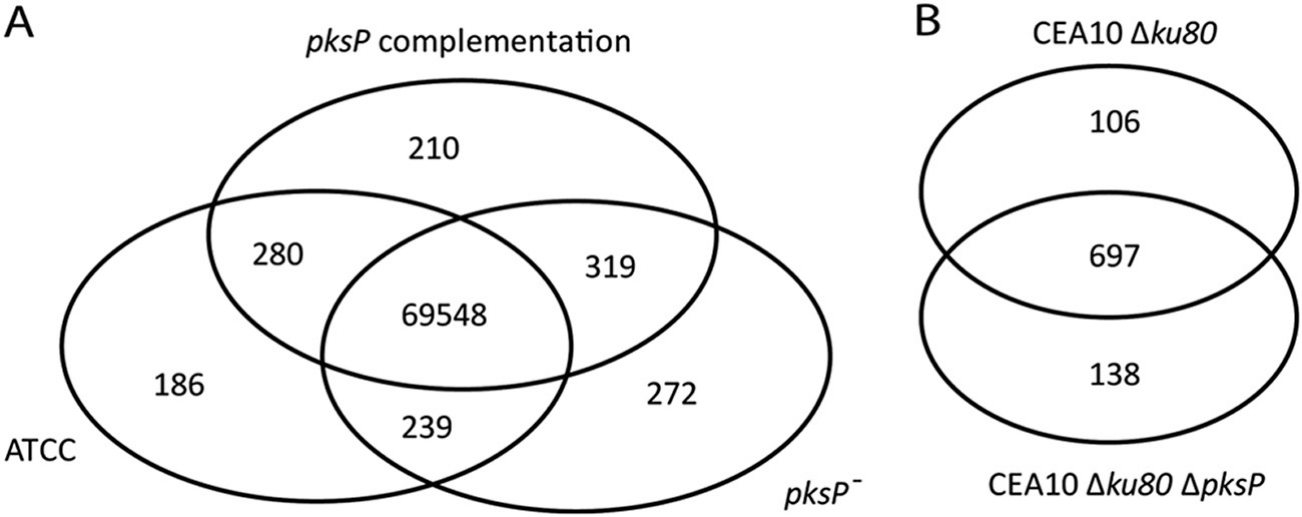
(A) Venn diagrams of the SNPs present in the sequenced ATCC strains mapped to the Af293 reference genome. (B) The SNPs of the sequenced CEA10 strains are mapped to the CEA10 reference genome.

**FIG 4:**
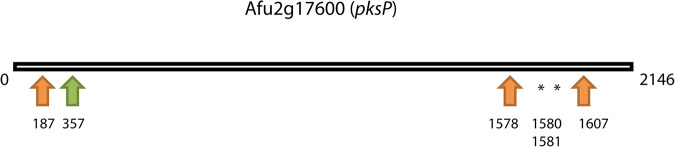
SNPs found in the ATCC *pksP^−^* mutant strain. Orange arrows indicate SNPs that lead to an amino acid change, the green arrow indicates an SNP with no amino acid change, and the asterisk indicates SNPs that lead to an amino acid deletion.

The ATCC 46645 wild-type strain, the derived *pksP* mutant strain, the *pksP* complemented mutant strain, and CEA10Δ*ku80* and CEA10Δ*ku80*Δ*pksP* strains were sequenced to assess whether SNPs in genes other than *pksP* can explain the increased H_2_O_2_ sensitivity of the *pksP^−^* strain and the increased superoxide resistance of the CEA10Δ*ku80*Δ*pksP* strain. Reads from the ATCC 46645 wild-type and derived strains were mapped back to the Af293 reference genome (30), while reads from the CEA10-derived strains were mapped back to the CEA10 reference genome (31). A total of 272 SNPs were unique to the ATCC 46645 *pksP^−^* strain (Fig. 3), of which 6 were in the *pksP* gene (Table 1, Fig. 4). This resulted in 3 amino acid changes and 2 missing amino acids due to deletion of codons, explaining the white phenotype of this strain. It is important to stress that complementation with the wild-type *pksP* gene resulted in normal pigmentation of conidia, indicating the absence of mutations in the other genes involved in DHN-melanin biosynthesis. Indeed, this was confirmed by the genome sequence. Next, we focused on SNPs in genes involved in ROS resistance (Table S2A) that were present in the ATCC 46645 *pksP^−^* strain and its *pksP*-complemented strain, which are both ROS sensitive and absent from the ATCC 46645 wild-type strain. An SNP in the *cat1* gene resulting in a change of lysine 719 to glutamic acid was present in both the mutant and complemented strain (Fig. S6). This change in charged amino acids might affect the function of the Cat1 protein, leading to an increase in H_2_O_2_ sensitivity.

**TABLE 1:** Amino acid changes resulting from SNPs in the *pksP* gene in ATCC 46645 *pksP^−^* mutant strain

Position	Amino acid change
187	Asparagine→aspartic acid
1578	Aspartic acid→asparagine
1580	Isoleucine deletion
1581	Isoleucine deletion
1607	Serine→phenylalanine

10.1128/msphere.00874-21.2 TABLE S2 (A) Genes involved in ROS protection of conidia and hyphae. (B) Genes of the BER pathway. Download Table S2, DOCX file, 0.03 MB. Copyright © 2022 Keizer et al. 2022 Keizer et al. https://creativecommons.org/licenses/by/4.0/ This content is distributed under the terms of the Creative Commons Attribution 4.0 International license.

10.1128/msphere.00874-21.8 FIG S6 PCR confirmation of the SNP in the *cat1* gene present in the ATCC*pksP^−^* and the ATCC *pksP* complementation strain. Download FIG S6, TIF file, 0.3 MB. Copyright © 2022 Keizer et al. 2022 Keizer et al. https://creativecommons.org/licenses/by/4.0/ This content is distributed under the terms of the Creative Commons Attribution 4.0 International license.

SNPs in the CEA10Δ*ku80* genome were also compared to the CEA10 reference genome, and three genes related to ROS sensitivity did contain an SNP. The first gene is AFUB_018600, and an ortholog in A. fumigatus (Afu2g01520) has been predicted to have a function in the oxidative stress response (https://fungidb.org). The second gene is AFUB_099260, which is predicted to play a role in oxidation-reduction processes (fungidb.org), whereas the third gene, AFUB_101360, is predicted to play a role in the cellular H_2_O_2_ response (fungidb.org). Mutations in these genes might be responsible for the decreased sensitivity of the CEA10Δ*ku80* strain for menadione, but this requires more research.

### Cat1 and peroxide sensitivity.

**FIG 5:**
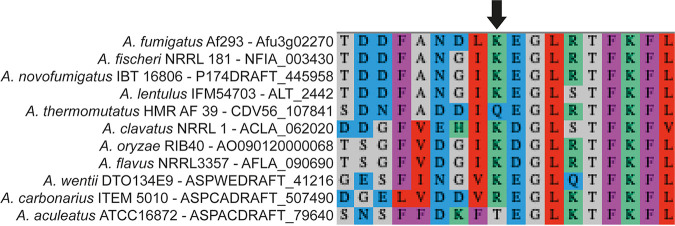
Top 10 blast results, based on E-value and score, of Cat1 protein sequence (Afu3g02270) within the aspergilli. The arrow indicates the amino acid that changed from a lysine (K) into a glutamic acid (E) in the ATCC 46645 *pksP^−^* mutant. Q, glutamine (polar uncharged side chain). R, arginine (positive charged side chain, just as lysine). T, threonine (polar uncharged side chain).

The observed SNP in the *cat1* gene leads to an amino acid change on position 719 from a lysine to a glutamic acid, positioned at the end of the protein. It is predicted that this part of the protein is involved in the interaction with calcium (32). A BLAST comparison with the top 10 results from the aspergilli shows that position 719 is not very variable (Fig. 5). Only in *A. thermomutatus* (CDV56_107841) and *A. aculeatus* (ASPACDRAFT_79640) is the amino acid changed from an amino acid with a positive-charged side chain to an amino acid with an uncharged side chain. The absence of variance at this location suggests that the structure of the part of the protein that interacts with calcium is important for its function.

**FIG 6:**
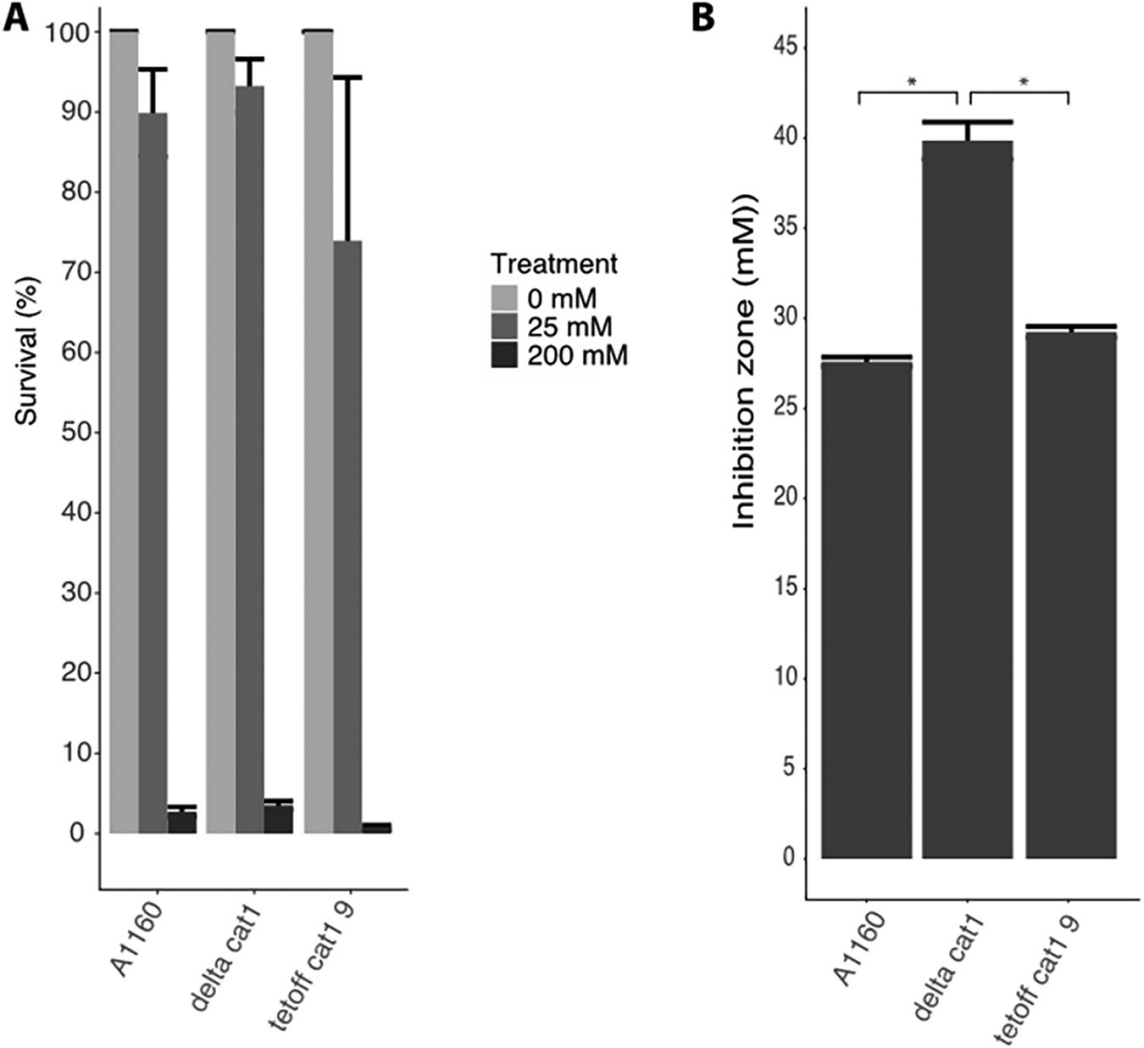
Sensitivity of dormant conidia to 0 mM, 25 mM, or 200 mM H_2_O_2_ (A) and mycelium toward 500 mM H_2_O_2_ (B) of A1160+ (wild-type), the Cat1 deletion strain, and its complementation strain (Tetoff Cat1). Bars represent the average inhibition zone based on biological and technical triplicates with the standard errors. Representative images can be found in Fig. S7.

To determine if the *cat1* gene product is involved in protection of the mycelium against H_2_O_2_, a *cat1* deletion mutant in an A1160 background and its complementation strain, as well as the wild-type strain, were compared in H_2_O_2_ stress experiments. As expected, dormant conidia of these three strains did not show any difference in sensitivity toward H_2_O_2_ (Fig. 6A), but when the growing mycelium of the strains was exposed to H_2_O_2_ in the plate assay, we indeed saw an increase in sensitivity toward H_2_O_2_ for the Cat1 deletion strain compared to the wild-type strain (A1160+) (Fig. 6B). This sensitivity is reversed when the *cat1* gene is reintroduced in the Cat1 deletion strain. These results show that the *cat1* gene is important in the protection of the mycelium against H_2_O_2_ stress.

## DISCUSSION

Here, it is shown by deletion of the *pksP* gene and its subsequent complementation that DHN-melanin does not protect against H_2_O_2_ and superoxides in strain CEA10 of A. fumigatus and strain LCP96 04111 of *Penicillium roqueforti.* These results are in contrast to results obtained with the ATCC 46645 *pksP^−^* strain (17, 18). Therefore, the latter strain was complemented by introducing *pksP* (33). This restored DHN-melanin formation but not H_2_O_2_ resistance. This implied that another mutation should be responsible for H_2_O_2_ sensitivity. Indeed, a mutation was found in the *cat1* gene encoding a secreted catalase. Catalases and peroxiredoxins play a role in the detoxification of H_2_O_2_ and are both regulated by Yap1 (26, 34). *Yap1* is identified as a master regulator of genes against ROS, and deletion of this transcription factor increases the sensitivity toward H_2_O_2_ and menadione (34). Other catalase genes in the A. fumigatus genome as well as the peroxiredoxin genes and the y*ap1* gene did not have mutations. In Fusarium oxysporium *yap1* is suggested, next to its role as master regulator of genes against ROS, to be involved as a regulator of the DNA damage response genes (35). One of the pathways involved in DNA damage repair is the base excision repair (BER) pathway, which might be affected. We therefore analyzed whether SNPs were present in genes in the BER pathway (Table 2) of A. fumigatus ATCC 46645 *pksP^−^* and its complemented strain as well as CEA10 ku80 strain. However, no mutations were found in genes involved in the BER pathway (Table 2), suggesting that defects in this DNA repair machinery do not contribute to increased sensitivity to hydrogen peroxide of the ATCC 46645-derived strains, but more research is required to rule this out completely. H_2_O_2_ experiments also showed that the mycelium of the *cat1* deletion strain is more sensitive to H_2_O_2_ than its wild-type and complementation strains (Fig. 6B). Furthermore, it must be emphasized that the inhibition zones visible on the H_2_O_2_ assay plates of the ATCC 46645 *pksP^−^* and the *pksP* complemented strain hardly contained air bubbles compared to their corresponding WT strain (Fig. S4A). This observation underscores that these former ATCC 46645-derived strains have a strongly reduced catalase activity.

**TABLE 2:** Primers used in this study

Name	Sequence
*Cat1*_confirm_PCR_fw	CGGTTCGGCAAGACTGTT
*Cat1*_confirm_PCR_rv	CTTTAGCCCGTCCGTCAG
AFUA_3G02270_FWD (BM3)	CCTGAGTGGCCGTTTATGCGTCTCACGTTCATCCCCAGC
AFUA_3G02270 _REV (BM4)	CGCCCTTGCTCACTTGAGCCAGCGTGCACTCTTCATAATC
pSK379_tetOFF_FWD (BM1)	CCTGAGTGGCCGTTTATGCGTCTCACGTTCATCCCCAGC
pSK379_tetOFF_REV (BM2)	AACGTGAGACGCATAAACGGCCACTCAGGCCGGTGATGT

10.1128/msphere.00874-21.6 FIG S4 Representative images of peroxide (A) and menadione (B) sensitivity used for measuring the inhibition zones displayed in Fig. 1. Download FIG S4, TIF file, 0.8 MB. Copyright © 2022 Keizer et al. 2022 Keizer et al. https://creativecommons.org/licenses/by/4.0/ This content is distributed under the terms of the Creative Commons Attribution 4.0 International license.

We therefore conclude that the increased sensitivity of the ATCC 46645 *pksP^−^* strain is explained by the mutation in the *cat1* gene. This gene plays a role in ROS resistance of mycelium and not in conidia but has no effect on virulence (25). We confirmed that Cat1 is indeed responsible for peroxide resistance of mycelium but not conidia under our experimental conditions. Furthermore, dormant conidia of the ATCC 46645 *pksP^−^* strain did not show increased sensitivity to ROS, in contrast to developing mycelium in the plate assays used, which can be explained by the observed K719E mutation in *cat1*. Thus, growing hyphae but not conidia of the ATCC 46645 *pksP^−^* strain have increased sensitivity to peroxide. These results are in accordance with previously published research (25). The minimal role for *cat1* in virulence could mean that there are compensatory mechanisms upon deletion, which still protect the fungus against the H_2_O_2_ produced by immune cells. Alternatively, or in addition, other mechanisms of protection against ROS in the host are more important, e.g., the superoxide dismutases and peroxiredoxins.

Melanins have previously been shown to protect against a variety of stress factors (36). The absence of melanin increases the sensitivity of fungal species, such as C. neoformans and A. nidulans, against ROS like H_2_O_2_ (19, 21). It must be noted that both fungal species actually produce l-DOPA melanin, whereas A. fumigatus produces DHN-melanin. These melanins differ in their chemical structure (Fig. S8). l-DOPA melanin contains a dihydroquinone group (two -OH groups attached to one ring structure), which can be converted to quinones and release 2 electrons, with which they quench ROS radicals (37). Next to the dihydroquinone structure there is also an indole structure (-NH group in one ring structure) present in l-DOPA melanin, which can also quench free radicals produced by the ROS (38). Neither of these structures are present in DHN-melanin, which could explain why this type of melanin does not confer protection against H_2_O_2_ and superoxide generated with menadione. The same seems to be the case for Asp-melanin. This type of pigment is produced by Aspergillus terreus instead of l-DOPA or DHN-melanin. Deletion of the genes encoding Asp-melanin, *melA* and *tyrP*, also does not alter the sensitivity toward H_2_O_2_ (39).

10.1128/msphere.00874-21.9 FIG S7 Representative images of peroxide sensitivity used for measuring the inhibition zones displayed in Fig. 6. Download FIG S7, TIF file, 0.3 MB. Copyright © 2022 Keizer et al. 2022 Keizer et al. https://creativecommons.org/licenses/by/4.0/ This content is distributed under the terms of the Creative Commons Attribution 4.0 International license.

10.1128/msphere.00874-21.10 FIG S8 Structure of DHN-melanin (A) and l-DOPA melanin (B) (adapted from Eisenman and Casadevall [49]). Download FIG S8, TIF file, 0.1 MB. Copyright © 2022 Keizer et al. 2022 Keizer et al. https://creativecommons.org/licenses/by/4.0/ This content is distributed under the terms of the Creative Commons Attribution 4.0 International license.

The CEA10Δ*pksPΔku80* deletion strain was more resistant to menadione than the CEA10*Δku80* strain, which could be due to mutation in still-unknown genes with a role in superoxide protection. Of interest, deletion of the *ku80* gene in the CEA10 background results in an increase in resistance toward menadione. ROS induces DNA damage; therefore, we expected that deletion of *ku80* would lead to a further decrease in menadione resistance, since this strain cannot repair DNA damage by nonhomologous end joining (40). It could be that inactivation of *ku80* was accompanied by a mutation resulting in decreased menadione sensitivity.

Taken together, we demonstrated that DHN-melanin does not protect against H_2_O_2_, which has long been recognized as an important protecting compound. Instead, we show a role for the mycelial catalase encoded by the *cat1* gene in protection of hyphae against extracellular H_2_O_2._ We propose that the absence of dihydroquinone and indol structure in DHN-melanin explains the difference with l-DOPA melanin, which can quench free radicals generated via ROS.

## MATERIALS AND METHODS

### Strains and culture conditions.

Strains used in this study are listed in Table 3, and an overview of the mutants strains used can be found in Fig. S1 in the supplemental material. A. fumigatus and *Penicillium roqueforti* were grown on potato dextrose agar (PDA; Difco) at 37°C and 25°C, respectively, for production of conidia. Conidia were harvested with 0.85% (wt/vol) NaCl with 0.005% Tween 20 (VWR International). A. fumigatus and *P. roqueforti* were grown on minimal medium (MM; 6 g L^−1^ NaNO_3_, 1.5 g L^−1^ KH_2_PO_4_, 0.5 g L^−1^ KCl, 0.5 g L^−1^ MgSO_4_·7H_2_O, 0.2 ml L^−1^ Vishniac, and 20 mM glucose) or transformation medium (TM; MM with 25 mM glucose, 5 g L^−1^ yeast extract [Difco], and 2 g L^−1^ Casamino Acids [Difco]) for genomic DNA isolation.

**TABLE 3:** Strains used in this study

Strain	Description	Reference or source
A. fumigatus		
ATCC 46645^*a*^	Aspergillus fumigatus, clinical isolate	17
ATCC 46645 *pksP^−^*^*a*^	*pksP^−^* derivative of ATCC 46645 obtained via UV mutagenesis	17
KL1.1^*a*^	*pksP^−^*::*pksP* in ATCC 46645*pksP* mutant	33
CEA10	Aspergillus fumigatus, clinical isolate	31
CEA10Δ*ku80*	*pyrG*::ku80 in CEA10	40
CEA10Δ*pksP*	Δ*pksP*::hph in CEA10Δ*KU80*	46
CEA10*pksP*C	*pksP*::*pksP* derivative of CEA10Δ*pksP*	46
A1160+	*ΔakuB*^KU80^; *pyrG*^+^	47
ΔAFUA_3G02270^*b*^	*ΔakuB^KU80^*; Δ*cat1*::hph in A1160+	This study
A1160+_tetOFF-*cat1*^*b*^	*ΔakuB^KU80^*; Δ*cat1*::hph,His2A::tetOFF-*cat1-ptrA*	This study
*P. roqueforti*		
LCP96 04111	*Penicillium roqueforti* wild-type	48
PT34.2	*pksA* mutant in LCP96 04111	28

a Strains were kindly provided by Axel A. Brakhage from HKI-Jena.

b Strains were kindly provided by Elaine M. Bignell from the University of Exeter.

10.1128/msphere.00874-21.3 FIG S1 Schematic overview of the background and constructed melanin (A) and catalase (B) mutant strains. Download FIG S1, TIF file, 0.1 MB. Copyright © 2022 Keizer et al. 2022 Keizer et al. https://creativecommons.org/licenses/by/4.0/ This content is distributed under the terms of the Creative Commons Attribution 4.0 International license.

### Peroxide and menadione sensitivity assays. (i) Plate stress assay.

Peroxide and menadione sensitivity assays were performed as described previously (18, 34). Briefly, 5 × 10^7^ A. fumigatus conidia were mixed with 5 ml MM agar without a carbon source and poured onto MM agar plates (6 cm in diameter, containing 10 ml MM medium). For *P. roqueforti*, 10^7^ conidia were mixed with 10 ml MM agar without carbon source and poured onto MM agar plates (10 cm in diameter, containing 20 ml MM medium). A hole of 10 mm was punched in the middle of the plate and filled with 100 μl 500 mM H_2_O_2_ (Sigma-Aldrich) or 100 μl 1 mM menadione (Sigma-Aldrich). Plates were incubated at 37°C for 16 h after which the inhibition zone was measured.

### (ii) Direct exposure stress assay.

Conidia were harvested from 3-day-old potato dextrose agar (PDA)-grown colonies, and resting conidia were diluted in Milli-Q to a concentration of 1 × 10^6^ ml^−1^ and incubated for 30 min at room temperature with 0, 25, or 200 mM H_2_O_2_. Conidia were subsequently diluted in Milli-Q to a concentration of 1 × 10^3^ ml^−1^, and 100 μl was plated onto PDA. The numbers of CFU were counted after overnight incubation at 37°C, and survival percentages were calculated. The number of colonies counted from the plate with 0 mM H_2_O_2_ was set as 100% survival.

### Chromosomal DNA isolation.

Conidia were inoculated in TM and 50 μg ml^−1^ ampicillin (Sigma-Aldrich) to prevent bacterial contamination and grown overnight at 37°C and 200 rpm. Mycelium was collected by filtering over a double layer of Miracloth (Merck Millipore) and lyophilized overnight. Part of the lyophilized mycelium (∼30 mg) was homogenized with a TissueLyser (Qiagen) using 2 metal balls (4.76 mm in diameter) for 2 min at 25 Hz. DNA was isolated from the homogenized mycelium with the Qiagen DNeasy PowerPlant Pro kit by following the manufacturer’s protocol for problematic samples. Qubit was used to check DNA quality and concentration. DNA samples were stored at −20°C.

### DNA sequencing and SNP analysis.

Whole-genome sequencing was performed by the Utrecht sequencing facility (USEQ). Libraries were prepared using a TruSeq DNA Nano library and sequenced on an Illumina NextSeq500 with 150-bp paired-end mid-output configuration. The quality of the reads was checked using fastQC (https://www.bioinformatics.babraham.ac.uk/projects/fastqc/). Cleaning and trimming of the reads was performed using the Fastx-Toolkit (http://hannonlab.cshl.edu/fastx_toolkit/). Reads were mapped to the genome of reference strain Af293 for the reads from the ATCC-derived strains (release 42 from FungiDB; https://fungidb.org/fungidb/) or reference strain CEA10 for the CEA10-derived strains (release 42 from FungiDB; https://fungidb.org/fungidb/) with bowtie2 v2.2.9 using options end to end and very sensitive (41). For further quality control, SAMtools v1.3 was used. Freebayes v.0.9.10-3 (42) with the ploidy option set to 1 was used for variant calling. Vcfilter (“qual > 20,” depth 5×) was used for postfiltering of the obtained vcf file. SNPeff v4.3 was used to predict the effect of the variants (43). SNPs with a high (gain or loss of a stop codon, splice region variant) or moderate (missense) effect were used for analysis.

### Cat1 SNP confirmation.

The SNP in the *cat1* gene of the ATCC 46645 *pksP^−^* mutant strain and the *pksP* complementation strain was confirmed by sequencing the *cat1* PCR product amplified with primers *Cat1*_confirm_PCR_fw and *Cat1*_confirm_PCR_rv (Table 2) and Q5 polymerase (New England Biolabs Inc.) according to the manufacturer’s instructions. The sequences of the PCR products were aligned with the ATCC *cat1* gene using the Clustal Omega web tool (https://www.ebi.ac.uk/Tools/msa/clustalo/).

The Cat1 protein sequence (AFUA_3G02270) was retrieved from fungidb.org, and homologous sequences were found via a protein-BLAST (using default setting provided by fungidb.org) search within the Aspergillus species and in A. fumigatus. The top 10 hits were selected based on E-value and score (Table S1). Retrieved sequences were aligned using the Mafft web tool (https://mafft.cbrc.jp/alignment/software/).

10.1128/msphere.00874-21.1 TABLE S1 Top 10 hits by a protein-protein BLAST, ranked on E-value and score, on *cat1* (Afu3g02270). Download Table S1, DOCX file, 0.03 MB. Copyright © 2022 Keizer et al. 2022 Keizer et al. https://creativecommons.org/licenses/by/4.0/ This content is distributed under the terms of the Creative Commons Attribution 4.0 International license.

### Cat1 complementation plasmid.

A1160+ was used to obtain the *cat1* (AFUA_3G02270) wild-type sequence by PCR using primers AFUA_3G02270_FWD and AFUA_3G02270 _REV (Table 2) and PhusionFlash high-fidelity polymerase (Thermo Fisher) according to the manufacturer’s instructions. Primers contain homology with the pSK606_tetOFF plasmid backbone (44) (Fig. S2A). The plasmid was linearized using primers pSK379_tetOFF_FWD and pSK379_tetOFF_REV. GeneArt Seamless Cloning technology (Thermo-Fisher) was used to generate the Cat1 complementation plasmid according to the manufacturer’s instructions. Correct integration of insert in the plasmid was confirmed by a PCR of the *cat1* gene using primers AFUA_3G02270_FWD and AFUA_3G02270 _REV and a PVUI restriction digest (New England Bio Labs [NEB]) according to the manufacturer’s instructions (Fig. S2B).

10.1128/msphere.00874-21.4 FIG S2 (A) Fragments (plasmid backbone) and *cat1* insert with their corresponding oligonucleotides for amplification (Table 2) and ligation using GeneArt Seamless cloning per the manufacturer’s instructions. Figure was made using Biorender.com. (B) Verification of plasmids by *cat1* PCR amplification (lanes 1 to 7, amplicon size 3502 bp) and PVU1 enzyme digestion (lanes 8 to 14). Download FIG S2, JPG file, 0.1 MB. Copyright © 2022 Keizer et al. 2022 Keizer et al. https://creativecommons.org/licenses/by/4.0/ This content is distributed under the terms of the Creative Commons Attribution 4.0 International license.

### Transformation A. fumigatus.

The Cat1 complementation plasmid was introduced into A. fumigatus ΔAFUA_3G02270 using a modified version of the method described previously (45), with pyrithiamine as a selection marker. Mycelia grown overnight in Aspergillus complete medium (ACM; 0.075 g L^−1^ adenine, 10 g L^−1^ glucose, 1 g L^−1^ yeast extract, 2 g L^−1^ bacteriological peptone, 1 g L^−1^ Casamino Acids, 10 ml L^−1^ vitamin solution [400 mg L^−1^ 4-aminobenzoic acid, 50 mg L^−1^ thiamine, 1 mg L^−1^ biotin, 24 g L^−1^ inositol, 100 mg L^−1^ nicotinic acid, 200 mg L^−1^ dl-phantothenic acid, 250 mg L^−1^ pyridoxine, 100 mg L^−1^ riboflavin, 1.4 g L^−1^ choline chloride], 20 ml L^−1^ salt solution, 10 ml L^−1^ 500 mM ammonium tartrate, set pH to 6.5 with 10 mM NaOH) (30°C, 150 rpm) was harvested and filtered through sterile Miracloth and resuspended in 40 ml protoplasting solution, containing 20 ml ACM, 20 ml 0.6 M KCl, citric acid solution, and 2.56 g of VinoTaste enzyme mix (NOVOZYMES), prior to incubation for 4 h (30°C, 150 rpm). Protoplasts were filtered using a 40-μm cell strainer (Corning) to remove mycelial debris, and solution was made up to a final volume of 50 ml with 0.6 M KCl and samples were centrifuged at 1,800 × *g* for 10 min. Pellets were resuspended in 2 ml 0.6 M KCl, transferred to two independent Eppendorf tubes, and centrifuged at 2,400 × *g* for 3 min prior to reprocessing in 0.6 M KCl a further three times. Pellets were then resuspended in 1 ml 0.6 M KCl, 50 mM CaCl_2_ and centrifuged at 2,400 × *g* for 3 min, pellets were resuspended in 500 μl 0.6 M KCl, 50 mM CaCl_2_ solution, and tubes were combined, pelleted at 2,400 × *g* for 3 min, and resuspended in a volume of 0.6 M KCl, 50 mM CaCl_2_ appropriate for the experiment (100 μl/condition). Volumes of 100 μl of protoplasts were then incubated with 10 ng of transforming DNA on ice for 25 min. Following ice incubation, 1 ml of 40% polyethylene glycol 3350 (Sigma) is added to the tube and incubated for a further 25 min at room temperature. Protoplast-DNA solution is then plated onto plates containing regeneration medium (1.5%, wt/vol, bacteriological agar no. 1 [Oxoid]) containing 500 ng μL^−1^ pyrithiamine via overlay with regeneration medium (0.6%, wt/vol, bacteriological agar no. 1). Transformation plates are left at RT overnight prior to incubation at 37°C for at least 3 days. Successful transformants were streaked onto MM plates containing pyrithiamine at least 2 times.

Prior to confirming correct integration of the Cat1 complementation plasmid in the ΔAFUA_3G02270 strain, resulting in A1160^+^_tetOFF-*cat1*, genomic DNA was isolated by resuspending conidia in 200 μl of DNA breaking buffer (2% Triton X-100, 1% SDS, 100 mM NaCl, 10 mM Tris-HCl pH 8.0, 1 mM EDTA, pH 8.0). Volumes of 300 mg of glass beads (0.4- to 0.6-mm diameter; Sigma) were added to samples, briefly vortexed, and incubated for 30 min at 70°C, with further vortexing every 10 min. A volume of 200 μl phenol-chloroform-isoamyl alcohol (25:24:1) (Sigma-Aldrich) was added prior to vortexing for 5 min and subsequently centrifuged for 8 min at 13,000 rpm to remove debris. A volume of 1 ml of isopropanol was added to the collected supernatant and incubated for 1 h at −20°C, followed by further centrifugation at 13,000 rpm for 10 min. The collected pellet was washed in 70% (vol/vol) ethanol (Sigma-Aldrich) and air dried before resuspension in 20 μl of sterile H_2_O. Correct integration was confirmed by PCR using primers AFUA_3G02270_FWD and AFUA_3G02270 _REV (Fig. S3).

10.1128/msphere.00874-21.5 FIG S3 Verification of *cat1* expression under the control of the tetOFF promotor in A. fumigatus by *cat1* PCR amplification. Possible successful transformants (lanes 1 to 10), tetOFF-*cat1* plasmid (positive control, lane 11), and *cat1* deletion strain (negative control, lane 12) are shown. Download FIG S3, TIF file, 0.04 MB. Copyright © 2022 Keizer et al. 2022 Keizer et al. https://creativecommons.org/licenses/by/4.0/ This content is distributed under the terms of the Creative Commons Attribution 4.0 International license.

### Statistical analysis.

Differences in inhibition zone after H_2_O_2_ or menadione exposure were analyzed using one-way analysis of variance (*P* ≤ 0.05). Differences in survival percentage of conidia after peroxide treatments were analyzed using a nonparametric Kruskal-Wallis test (*P* ≤ 0.05). Bars represent the average inhibition zone or survival percentage based on biological and technical triplicates with the standard errors.

### Data availability.

Next-generation sequencing (NGS) data of strains ATCC 46645 and CEA10Δ*ku80* are available at the NCBI Sequence Read Archive (SRA) under code PRJNA670081. NGS data from ATCC 46645 *pksP^−^*, KL1.1, and CEA10Δ*pksP* strains are available from the NCBI SRA under code PRJNA680589.
