# Supplementary figures and images for: The Protective Role of 1,8-Dihydroxynaphthalene–Melanin on Conidia of the Opportunistic Human Pathogen Aspergillus fumigatus Revisited: No Role in Protection against Hydrogen Peroxide and Superoxides
Source: mSphere. 2022 Jan 5;7(1):e00874-21. doi: 10.1128/msphere.00874-21 (PMC8730813; doi:10.1128/msphere.00874-21)

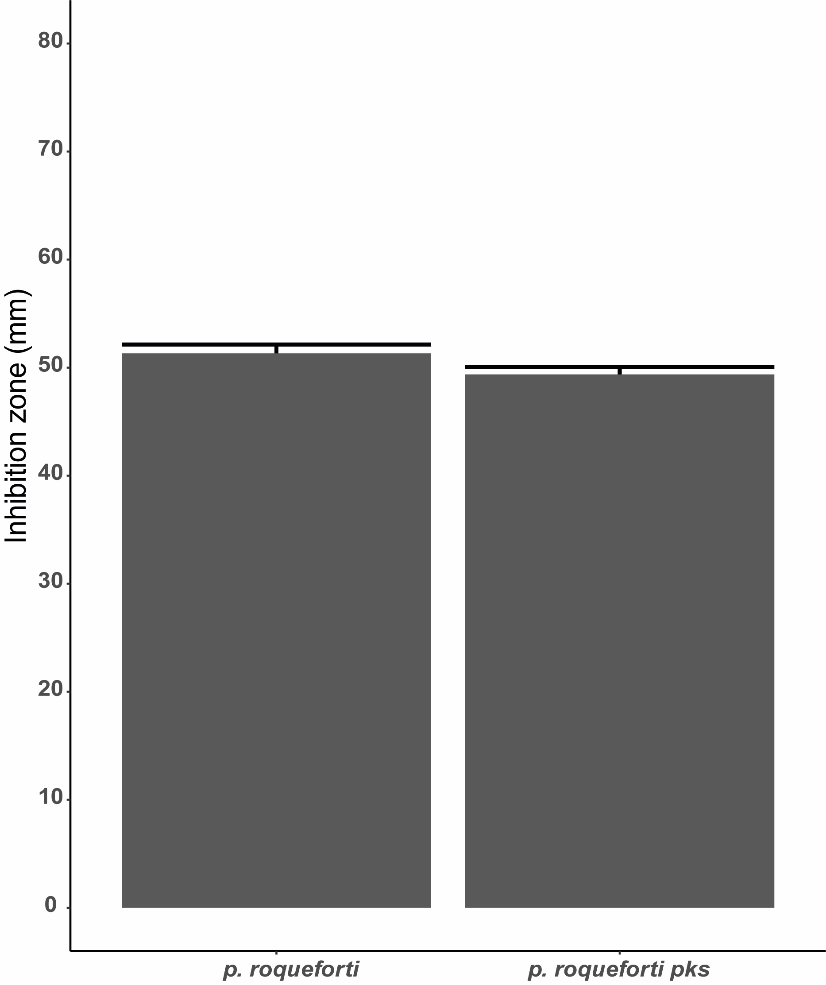

Supplement: FIG S5 [file msphere.00874-21-sf005.tif]

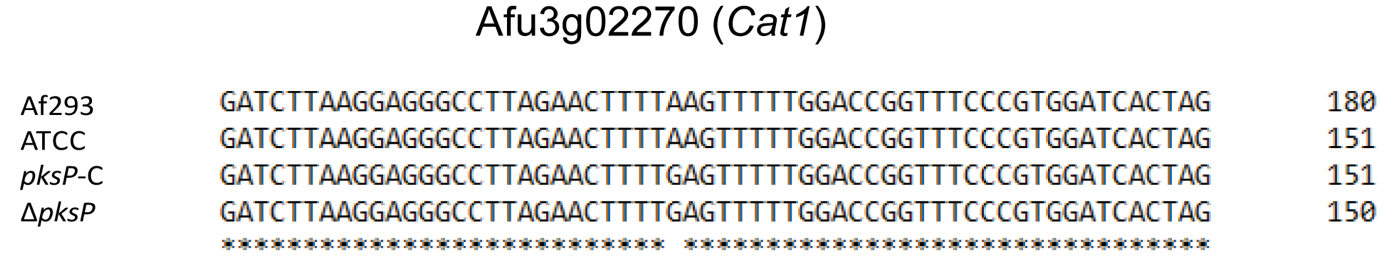

Supplement: FIG S6 [file msphere.00874-21-sf006.tif]

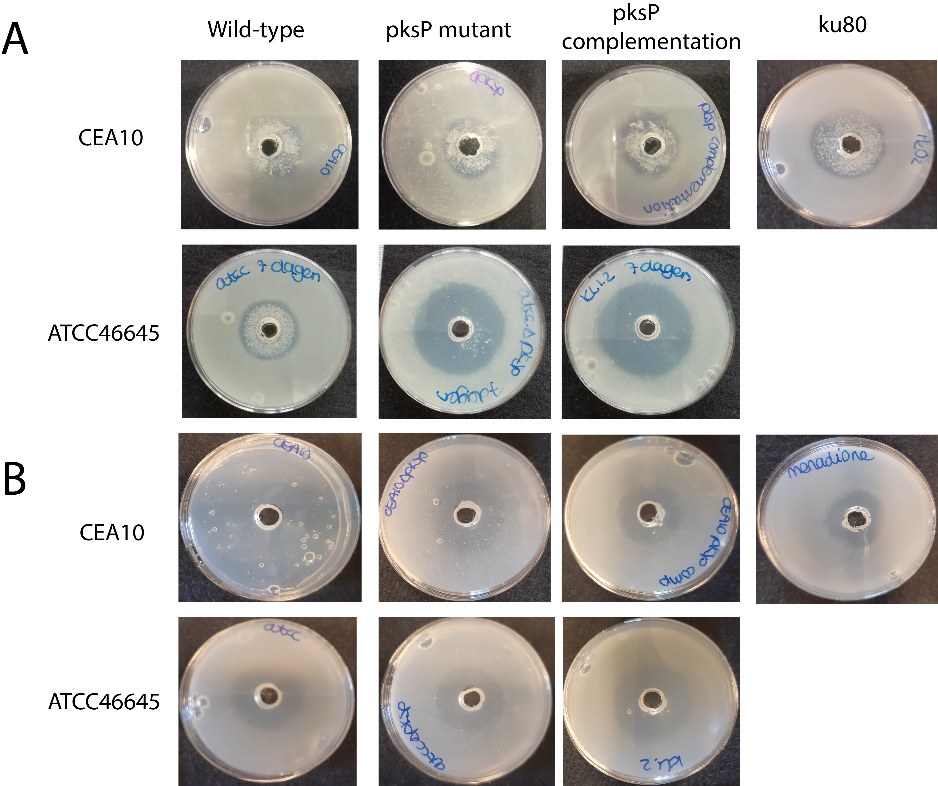

Supplement: FIG S4 [file msphere.00874-21-sf004.tif]

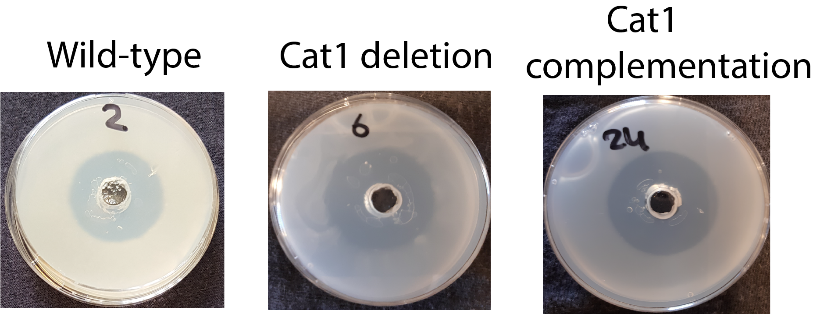

Supplement: FIG S7 [file msphere.00874-21-sf007.tif]

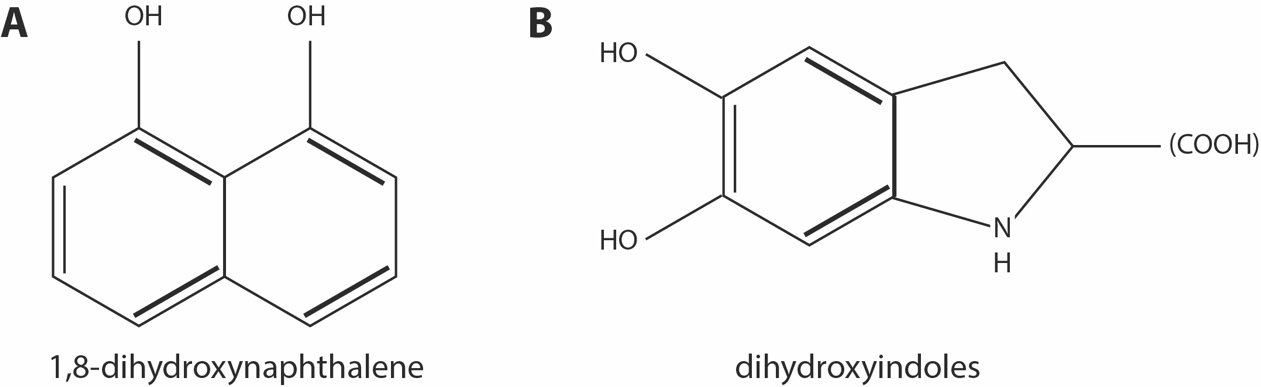

Supplement: FIG S8 [file msphere.00874-21-sf008.tif]

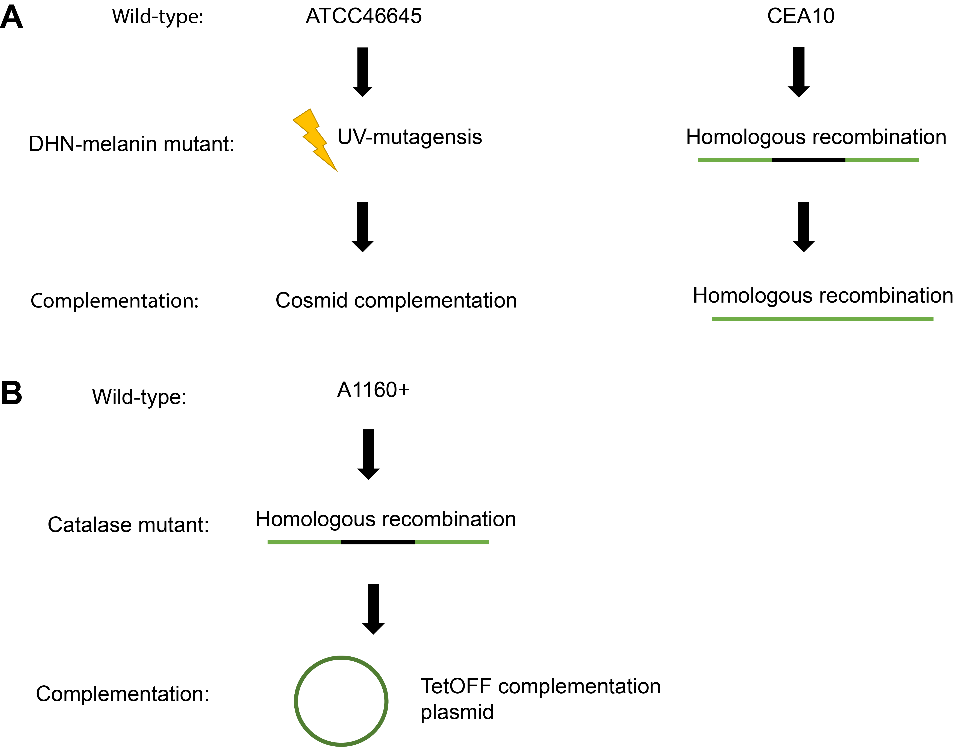

Supplement: FIG S1 [file msphere.00874-21-sf001.tif]

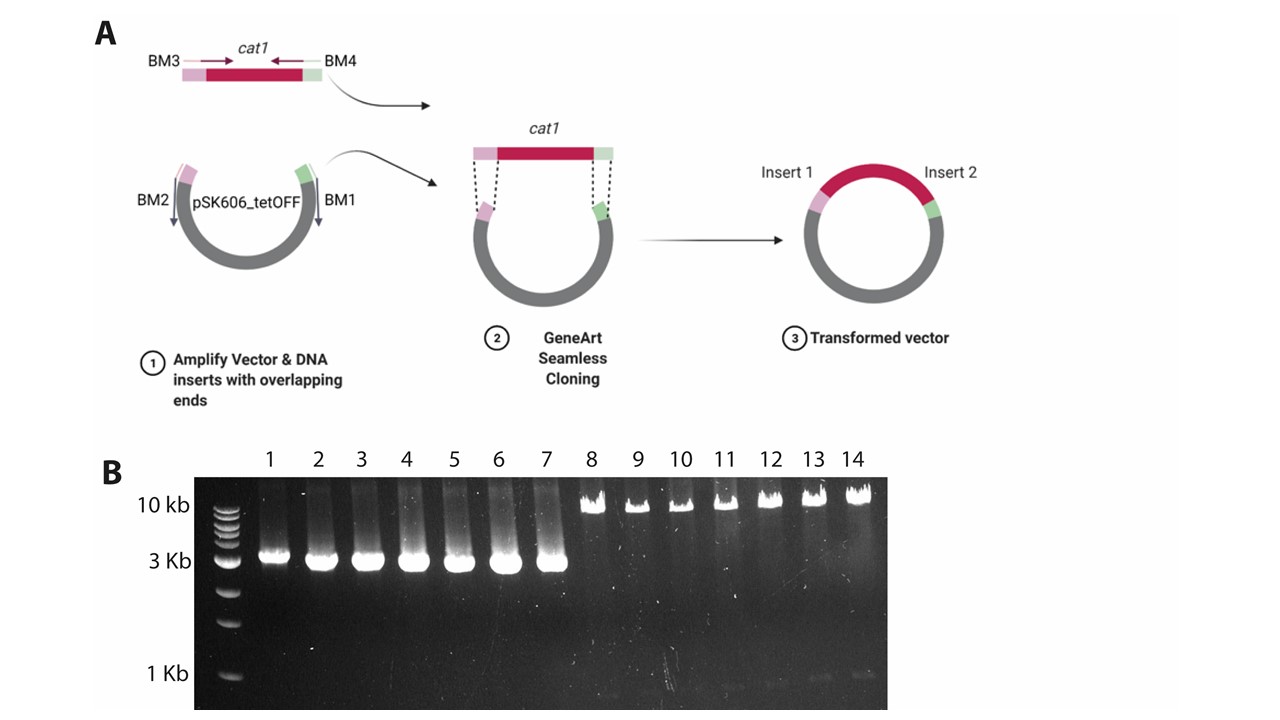

Supplement: FIG S2 [file msphere.00874-21-sf002.jpg]
