# Supplementary material for: The Protective Role of 1,8-Dihydroxynaphthalene–Melanin on Conidia of the Opportunistic Human Pathogen Aspergillus fumigatus Revisited: No Role in Protection against Hydrogen Peroxide and Superoxides
Source: mSphere. 2022 Jan 5;7(1):e00874-21. doi: 10.1128/msphere.00874-21 (PMC8730813; doi:10.1128/msphere.00874-21)
Supplement: TABLE S2 [file msphere.00874-21-st002.docx]

**Supplementary Table 2A.** Genes involved in ROS protection of conidia and hyphae.

| Gene | | GeneID | | Function | Reference |  |
| --- | --- | --- | --- | --- | --- | --- |
| *yap1* | | Afu6g09930 | | Transcription factor that regulates catalase expression | (1) |  |
| *catA* | | Afu6g03890 | | Conidial catalase | (2) |  |
| *cat1* | | Afu3g02270 | | Mycelium catalase | (2,3) |  |
| *cat2* | | Afu8g01670 | | Mycelium catalase | (2) |  |
| *pksP* | | Afu2g17600 | | DHN-melanin synthesis | (4,5) |  |
| *aspf3* | | Afu6g02280 | | Fungal allergen and has peroxidase activity | (6) |  |
| *rgsD* | | Afu5g00900 | | Regulator of G protein signaling | (7) |  |
| *aox* | | Afu2g05060 | | Mitochondrial enzyme alternative oxidase. Decreases damage caused by oxidative stress / minimize production ROS | (8) |  |
| *cycA* | | Afu2g13110 | | Cytochrome C, part of the respiratory chain to generate ATP | (8) |  |
| *pab1* | | Afu1g04190 | | Cytoplasmic messenger ribonuclear protein | (9) |  |
| *sod1 (A)* | | Afu5g09240 | | Superoxide dismutase, expressed in conidia, Cytoplasmic Cu/Zn SOD | (10) |  |
| *sod2 (B)* | | Afu4g11580 | | Superoxide dismutase, expressed in conidia, Mitochondrial Mn SOD | (10) |  |
| *prx1* | | Afu4g08580 | | Cytosolic peroxiredoxin | (11) |  |
| *prxB* | | Afu5g15070 | | Mitochondrial peroxiredoxin | (11) |  |
| *prxC* | | Afu8g07130 | | Mitochondrial peroxiredoxin | (11) |  |
| *gstA* | | Afu3g10830 | | Glutathione peroxidase | (12) |  |
| *gstC* | | Afu4g14530 | | Glutathione peroxidase | (12) |  |
| *sakA* | | Afu1g12940 | | Putative mitogen-activated protein kinase (MAPK) with predicted roles in the osmotic and oxidative stress responses | (13) |  |
| *phkA* | | Afu3g12550 | | Class X histidine kinase | (13) |  |
| *atfA* | | Afu3g11330 | | bZIP-type transcription factor | (13) |  |
| *bir1* | | Afu1g14070 | | Inhibitor of apoptosis protein 1 | (14) |  |
| *hyr1* | Afu3g12270 | | Putative glutathione peroxidase; peroxiredoxin | | (15) | |
| *skn7* | Afu6g12522 | | Putative transcription factor and response regulator of a two-component signal transduction system | | (16) | |
| *mpkA* | Afu4g13720 | | Mitogen-activated protein kinase | | (17) | |
| *mpkC* | Afu5g09100 | | Putative mitogen activated protein kinase (MAPK) | | (18) | |
| *pkaR* | Afu3g10000 | | cAMP-dependent protein kinase regulatory subunit | | (19) | |
| *gliK* | Afu6g06700 | | Gamma-glutamyl cyclotransferase gliK | | (20) | |
| *sskA* | Afu5g08390 | | Putative response regulator, part of a two-component signal transduction system | | (21) | |
| *pbsB* | Afu3g05900 | | MAP kinase kinase (MAPKK) | | (21) | |
| *sho1* | Afu5g08420 | | Putative transmembrane osmosensor with homology to S. cerevisiae Sho1p | | (22) | |
| *rho1* | Afu6g06900 | | Putative Rho-type GTPase | | (23) | |
| *pkcA* | Afu5g11970 | | Protein kinase C, involved in cell wall integrity pathway | | (24) | |
| *sebA* | Afu4g09080 | | putative transcription factor | | (25) | |

**Table 2B.** Genes of the BER pathway

| **Genes BER Pathway^1^** |  |  | AF gene |  |  | ATCC pksp + complementation | CEA10 ku80 |  |
| --- | --- | --- | --- | --- | --- | --- | --- | --- |
| short patch BER-pathway | Bifunctional glycolases | ogg1 | Afu7g05320 | AFUA_7G05320 |  | No SNP | AFUB_090900 | No SNP |
|  |  | NTH | Afu2g01120 | AFUA2_G01120 |  | No SNP | AFUB_018200 | No SNP |
|  |  | Fpg | Afu4g11930 | AFUA_4G11930 |  | No SNP | AFUB_068930 | No SNP |
|  | AP-endonuclease | APE1 | Afu6g08110 | AFUA_6G08110 |  | No SNP | AFUB_074090 | No SNP |
|  |  | APE2 | Afu3g06180 | AFUA_3G06180 |  | No SNP | AFUB_042880 | No SNP |
|  | Gap filling | lig | Afu2g09010 | AFUA_2G09010 |  | No SNP | AFUB_024900 | No SNP |
| Long path BER-pathway | monofunctional glycosylases | UNG | Afu2g06140 | AFUA_2G06140 |  | No SNP | AFUB_023220 | No SNP |
|  |  | AlkA | Afu4g06800 | AFUA_4G06800 |  | No SNP | AFUB_063870 | No SNP |
|  |  | TDG | Afu6g13340 | AFUA_6G13340 |  | No SNP | AFUB_001430 | No SNP |
|  | Strand scission | PCNA | Afu1g04900 | AFUA_1G04900 |  | No SNP | AFUB_005270 | No SNP |
|  |  | Pole | Afu2g06190 | AFUA_2G06190 |  | No SNP | AFUB_023270 | No SNP |
|  |  | Pol sigma | Afu2g16600 | AFUA_2G16600 |  | No SNP | AFUB_032280 | No SNP |
|  | Gap filling and strand displacement | Fen1 | Afu3g06060 | AFUA_3G06060 |  | No SNP | AFUB_042990 | No SNP |
| Ber complex | Short patch BER | APEX | Afu3g06180 | AFUA_3G06180 |  | No SNP | AFUB_042880 | No SNP |
|  |  | PARP | Afu5g07320 | AFUA_5G07320 |  | No SNP | AFUB_054870 | No SNP |

^1^ Genes in the BER pathway in *A. fumigatus* as described in <https://www.kegg.jp/pathway/afm03410>.

**References**

(1) Lessing F, Kniemeyer O, Wozniok I, Loeffler J, Kurzai O, Haertl A, et al. The Aspergillus fumigatus transcriptional regulator AfYap1 represents the major regulator for defense against reactive oxygen intermediates but is dispensable for pathogenicity in an intranasal mouse infection model. Eukaryotic Cell 2007 Dec;6(12):2290-2302.

(2) Paris S, Wysong D, Debeaupuis J, Shibuya K, Philippe B, Diamond RD, et al. Catalases of Aspergillus fumigatus. Infect Immun 2003 Jun;71(6):3551-3562.

(3) Calera JA, Paris S, Monod M, Hamilton AJ, Debeaupuis JP, Diaquin M, et al. Cloning and disruption of the antigenic catalase gene of Aspergillus fumigatus. Infect Immun 1997 Nov;65(11):4718-4724.

(4) Jahn B, Koch A, Schmidt A, Wanner G, Gehringer H, Bhakdi S, et al. Isolation and characterization of a pigmentless-conidium mutant of Aspergillus fumigatus with altered conidial surface and reduced virulence. Infect Immun 1997 Dec;65(12):5110-5117.

(5) Sugareva V, Härtl A, Brock M, Hübner K, Rohde M, Heinekamp T, et al. Characterisation of the laccase-encoding gene abr2 of the dihydroxynaphthalene-like melanin gene cluster of Aspergillus fumigatus. Arch Microbiol 2006 Nov;186(5):345-355.

(6) Hillmann F, Bagramyan K, Straßburger M, Heinekamp T, Hong TB, Bzymek KP, et al. The Crystal Structure of Peroxiredoxin Asp f3 Provides Mechanistic Insight into Oxidative Stress Resistance and Virulence of Aspergillus fumigatus. Sci Rep 2016 09 14,;6:33396.

(7) Kim Y, Lee M, Jun S, Choi Y, Yu J, Shin K. RgsD negatively controls development, toxigenesis, stress response, and virulence in Aspergillus fumigatus. Sci Rep 2019 01 28,;9(1):811.

(8) Grahl N, Dinamarco TM, Willger SD, Goldman GH, Cramer RA. Aspergillus fumigatus mitochondrial electron transport chain mediates oxidative stress homeostasis, hypoxia responses and fungal pathogenesis. Mol Microbiol 2012 Apr;84(2):383-399.

(9) Wang D, Wang S, He D, Gao S, Xue B, Wang L. Deletion of afpab1 Causes Increased Sensitivity to Oxidative Stress and Hypovirulence in Aspergillus fumigatus. Int J Mol Sci 2016 Oct 29,;17(11).

(10) Lambou K, Lamarre C, Beau R, Dufour N, Latge J. Functional analysis of the superoxide dismutase family in Aspergillus fumigatus. Mol Microbiol 2010 Feb;75(4):910-923.

(11) Rocha MC, de Godoy KF, Bannitz-Fernandes R, Fabri, João H. T. Marilhano, Barbosa MMF, de Castro PA, et al. Analyses of the three 1-Cys Peroxiredoxins from Aspergillus fumigatus reveal that cytosolic Prx1 is central to H2O2 metabolism and virulence. Sci Rep 2018 08 17,;8(1):12314.

(12) Burns C, Geraghty R, Neville C, Murphy A, Kavanagh K, Doyle S. Identification, cloning, and functional expression of three glutathione transferase genes from Aspergillus fumigatus. Fungal Genet Biol 2005 Apr;42(4):319-327.

(13) Day AM, Quinn J. Stress-Activated Protein Kinases in Human Fungal Pathogens. Front Cell Infect Microbiol 2019;9:261.

(14) Shlezinger N, Irmer H, Dhingra S, Beattie SR, Cramer RA, Braus GH, et al. Sterilizing immunity in the lung relies on targeting fungal apoptosis-like programmed cell death. Science 2017 09 08,;357(6355):1037-1041.

(15) Fan Z, Yu H, Guo Q, He D, Xue B, Xie X, et al. Identification and characterization of an anti-oxidative stress-associated mutant of Aspergillus fumigatus transformed by Agrobacterium tumefaciens. Mol Med Rep 2016 Mar;13(3):2367-2376.

(16) Lamarre C, Ibrahim-Granet O, Du C, Calderone R, Latgé J. Characterization of the SKN7 ortholog of Aspergillus fumigatus. Fungal Genet Biol 2007 Jul;44(7):682-690.

(17) Valiante V, Heinekamp T, Jain R, Härtl A, Brakhage AA. The mitogen-activated protein kinase MpkA of Aspergillus fumigatus regulates cell wall signaling and oxidative stress response. Fungal Genet Biol 2008 May;45(5):618-627.

(18) Bruder Nascimento, Ariane Cristina Mendes de Oliveira, Dos Reis TF, de Castro PA, Hori JI, Bom VLP, de Assis LJ, et al. Mitogen activated protein kinases SakA(HOG1) and MpkC collaborate for Aspergillus fumigatus virulence. Mol Microbiol 2016 06;100(5):841-859.

(19) Zhao W, Panepinto JC, Fortwendel JR, Fox L, Oliver BG, Askew DS, et al. Deletion of the regulatory subunit of protein kinase A in Aspergillus fumigatus alters morphology, sensitivity to oxidative damage, and virulence. Infect Immun 2006 Aug;74(8):4865-4874.

(20) Gallagher L, Owens RA, Dolan SK, O'Keeffe G, Schrettl M, Kavanagh K, et al. The Aspergillus fumigatus protein GliK protects against oxidative stress and is essential for gliotoxin biosynthesis. Eukaryotic Cell 2012 Oct;11(10):1226-1238.

(21) Hagiwara D, Suzuki S, Kamei K, Gonoi T, Kawamoto S. The role of AtfA and HOG MAPK pathway in stress tolerance in conidia of Aspergillus fumigatus. Fungal Genet Biol 2014 Dec;73:138-149.

(22) Ma Y, Qiao J, Liu W, Wan Z, Wang X, Calderone R, et al. The sho1 sensor regulates growth, morphology, and oxidant adaptation in Aspergillus fumigatus but is not essential for development of invasive pulmonary aspergillosis. Infect Immun 2008 Apr;76(4):1695-1701.

(23) Zhang X, Jia X, Tian S, Zhang C, Lu Z, Chen Y, et al. Role of the small GTPase Rho1 in cell wall integrity, stress response, and pathogenesis of Aspergillus fumigatus. Fungal Genet Biol 2018 11;120:30-41.

(24) Rocha MC, Godoy KFd, de Castro PA, Hori JI, Bom VLP, Brown NA, et al. The Aspergillus fumigatus pkcA G579R Mutant Is Defective in the Activation of the Cell Wall Integrity Pathway but Is Dispensable for Virulence in a Neutropenic Mouse Infection Model. PLoS ONE 2015;10(8):e0135195.

(25) Dinamarco TM, Almeida RS, de Castro PA, Brown NA, dos Reis TF, Ramalho LNZ, et al. Molecular characterization of the putative transcription factor SebA involved in virulence in Aspergillus fumigatus. Eukaryotic Cell 2012 Apr;11(4):518-531.
