# Supplementary material for: The Protective Role of 1,8-Dihydroxynaphthalene–Melanin on Conidia of the Opportunistic Human Pathogen Aspergillus fumigatus Revisited: No Role in Protection against Hydrogen Peroxide and Superoxides
Source: mSphere. 2022 Jan 5;7(1):e00874-21. doi: 10.1128/msphere.00874-21 (PMC8730813; doi:10.1128/msphere.00874-21)
Supplement: TABLE S1 [file msphere.00874-21-st001.docx]

**Supplementary Table 1.** Top 10 hits by a protein-protein BLAST, ranked on E-value and Score, on *cat1* (Afu3g02270)

| Gene ID | source_id | Organism | Score | E-Value |
| --- | --- | --- | --- | --- |
| NFIA_003430 | NFIA_003430_t1 | Aspergillus fischeri NRRL 181 | 1444 | 0,00E+00 |
| P174DRAFT_445958 | P174DRAFT_445958-t37_1 | Aspergillus novofumigatus IBT 16806 | 1441 | 0,00E+00 |
| ALT_2442 | ALT_2442_t1 | Aspergillus lentulus strain IFM 54703 | 1429 | 0,00E+00 |
| CDV56_107841 | CDV56_107841_t1 | Aspergillus thermomutatus strain HMR AF 39 | 1391 | 0,00E+00 |
| ACLA_062020 | ACLA_062020-t26_1 | Aspergillus clavatus NRRL 1 | 1342 | 0,00E+00 |
| AO090120000068 | AO090120000068-T | Aspergillus oryzae RIB40 | 1271 | 0,00E+00 |
| AFLA_090690 | AFLA_090690-t26_1 | Aspergillus flavus NRRL3357 | 1268 | 0,00E+00 |
| ASPCADRAFT_507490 | ASPCADRAFT_507490-t33_1 | Aspergillus carbonarius ITEM 5010 | 1231 | 0,00E+00 |
| ASPACDRAFT_79640 | ASPACDRAFT_79640-t33_1 | Aspergillus aculeatus ATCC 16872 | 1225 | 0,00E+00 |
| ASPWEDRAFT_41216 | ASPWEDRAFT_41216-t33_1 | Aspergillus wentii DTO 134E9 | 1225 | 0,00E+00 |
